# Characterizing and forecasting neoantigens-resulting from MUC mutations in COAD

**DOI:** 10.1186/s12967-024-05103-z

**Published:** 2024-03-27

**Authors:** Min Chen, Xin Zhang, Zihe Ming, Lingyu, Xiaorong Feng, Zhenguo Han, Han-Xiang An

**Affiliations:** 1 Clinical Central Research Core, Shanxi Bethune Hospital, Shanxi Medical University, Taiyuan, Shanxi China; 2 The Center Laboratory, Shanghai Medical College, Zhongshan Hospital (Xiamen Affiliated) of Fudan University, Fudan University, Xiamen, China; 3 Cancer Center and Department of Breast and Thyroid Surgery, School of Medicine, Xiang’an Hospital of Xiamen University, Xiamen University, Xiamen, China; 4 Shanxi Bethune Hospital, Shanxi Medical University, Taiyuan, Shanxi China; 5 Department of Chemistry and Key Laboratory for Preparation and Application of Ordered Structural Materials of Guangdong Province, Chemistry and Chemical Engineering Guangdong Laboratory, Shantou University, Guangdong, China; 6 Department of Colorectal and Anal Surgery, Shanxi Bethune Hospital, Shanxi Medical University, Taiyuan, Shanxi China; 7 The Cancer Center, Shanxi Bethune Hospital, Shanxi Medical University, Taiyuan, Shanxi China

**Keywords:** MUC, Tumor neoantigens, Colon adenocarcinoma, Bioinformatics, Immunotherapy

## Abstract

**Background:**

The treatment for colon adenocarcinoma (COAD) faces challenges in terms of immunotherapy effectiveness due to multiple factors. Because of the high tumor specificity and immunogenicity, neoantigen has been considered a pivotal target for cancer immunotherapy. Therefore, this study aims to identify and predict the potential tumor antigens of MUC somatic mutations (MUCmut) in COAD.

**Methods:**

Three databases of TCGA, TIMER2.0, and cBioPortal were used for a detailed evaluation of the association between MUCmut and multi-factors like tumor mutation burden (TMB), microsatellite instability (MSI), prognosis, and the tumor microenvironment within the context of total 2242 COAD patients. Next, TSNAdb and the differential agretopicity index (DAI) were utilized to predict high-confidence neopeptides for MUCmut based on 531 COAD patients’ genomic information. DAI was calculated by subtraction of its predicted HLA binding affinity of the MUCmut peptide from the corresponding wild-type peptide.

**Results:**

The top six mutation frequencies (14 to 2.9%) were from MUC16, MUC17, MUC5B, MUC2, MUC4 and MUC6. COAD patients with MUC16 and MUC4 mutations had longer DFS and PFS. However, patients with MUC13 and MUC20 mutations had shorter OS. Patients with the mutation of MUC16, MUC5B, MUC2, MUC4, and MUC6 exhibited higher TMB and MSI. Moreover, these mutations from the MUC family were associated with the infiltration of diverse lymphocyte cells and the expression of immune checkpoint genes. Through TSNAdb 1.0/NetMHCpan v2.8, 452 single nucleotide variants (SNVs) of MUCmut peptides were identified. Moreover, through TSNAdb2.0/NetMHCpan v4.0, 57 SNVs, 1 Q-frame shift (TS), and 157 short insertions/deletions (INDELs) of MUCmut were identified. Finally, 10 high-confidence neopeptides of MUCmut were predicted by DAI.

**Conclusions:**

Together, our findings establish the immunogenicity and therapeutic potential of mutant MUC family-derived neoantigens. Through combining the tools of TSNAdb and DAI, a group of novel MUCmut neoantigens were identified as potential targets for immunotherapy.

**Supplementary Information:**

The online version contains supplementary material available at 10.1186/s12967-024-05103-z.

## Background

Colon adenocarcinoma (COAD), the most prevalent form of colorectal cancer (CRC), ranks as the third most commonly diagnosed malignant tumor with a high mortality rate worldwide [1, 2]. Immunotherapy for cancer, like immune-checkpoint inhibitors (ICIs) that boost the body's natural anti-tumor immune responses, has demonstrated potential in treating recurrent or metastatic cancer [3–5]. The metastatic CRC patients with deficient mismatch repair (dMMR) or high microsatellite instability (MSI-H) receive a clear clinical response [6, 7], while those with proficient mismatch repair (pMMR)/microsatellite instability-low (MSI-L) CRC did not benefit from immunotherapy [8]. During the past 10 years, neoantigens, a class of antigens derived from tumor-specific mutations, have been widely regarded as optimal targets for activating the patient's immune system to recognize and eliminate cancer cells such as the mutations of KRAS [9], HER2 [10], and PIK3CA [11].

It has emerged as the most prevalent approach for the precise elimination of tumors through immunotherapy. Prof. Rosenberg’s research group reported the identification rate of neoantigen reactive T lymphocytes in gastrointestinal cancer was high (83%–90%) [12, 13] and also found that most epithelial cancers, generally considered to be non-immunogenic, do elicit in vivo immune reactions. Their studies offer a rationale for developing new immunotherapeutic treatments by targeting the unique tumor-associated mutations expressed in epithelial cancer. With the promising outcome from the neoantigen-based immunotherapies [14–19], researchers and clinicians are exploring the possibility of using tumor neoantigens as a potentially effective treatment to improve the treatment options for CRC patients.

In pan-cancer studies, a peptide-based neoantigen vaccine, iNeo-Vac-P01, was conducted on Chinese patients with solid tumors (NCT03662815) and showed a promising outcome in 30 patients, and 80% of peptides enhanced immune response [20, 21]. In a recent study (NCT03639714), the integration of neoantigen cancer vaccines GRT-C901 and GRT-R902 (produced by Gritstone Bio) with nivolumab and ipilimumab drugs was shown to significantly improve overall survival rates among patients with NSCLC, metastatic urothelial, gastroesophageal, and microsatellite stable colorectal cancers [21]. In a phase I/IIa clinical trial (NCT01461148) including 22 dMMR CRC patients treated with a united neoantigen-peptide containing three frameshift mutant genes (AIM2, HT001, TAF1B), two of the three assessable patients hold stable disease as the best overall response, and one heavily pretreated patient with bulky metastasis also presented as stable disease over 7 months [22]. Overall, the promising results of these results provided clinical prospects in terms of neoantigens-based immunotherapy in solid cancer.

The identification of immunogenic neoantigenes from numerous sources is a crucial step in the development of effective immunotherapies [23]. Neoantigens may now be thoroughly screened across the entire cancer spectrum thanks to the convergence of whole-exome sequencing (WES), RNA-seq, and proteomic data from TCGA [24]. Several studies showed a remarkable number of somatic mutations and tens of putative mutational neoantigens in COAD [25, 26], resulting in a vast total number of potential multiple neopeptides. To verify whether an antigen can be presented by the major histocompatibility complex (MHC), two general methods are used: (1) in silico computational predictions and (2) mass spectrum analyses. Several software programs are available for in silico computational predictions, including TSNAdb [27], NetMHCpan [28, 29], and IEDB [30]. The main principle of in silico computational predictions is to establish a predictive model through a database acquired by proteasome splicing, transporter associated with antigen processing (TAP) channel selectivity, and epitopes through which the MHC molecules recognize the peptide. Thus, ongoing attempts or strategies to improve neo-peptide immunogenicity prediction are critical for the selection of neoantigen targets for cancer.

The MUC family characterized by its heavily glycosylated transmembrane mucins has the same protein sequence in normal and tumor cells but different glycosylation profiles, thus the glycopeptide epitopes are expected to be tumor-specific. Studies showed that MUC1 neoantigens were exclusively recognized by glycoform-specific T cell receptors (TCR) [31] or presented on MHC class II molecules [32, 33]. Especially in recent years, MUC1 neoantigens has been proven as a tumor-associated antigen highly expressed on many adenocarcinomas (breast, colon, lung, kidney, ovary, etc.), and MUC1 neoantigens has been successfully utilized in immunotherapeutic approaches for the development of peptide-, carbohydrate-, DNA-, and dendritic cell (DC)-based vaccines [33], and clinical trials (NCT00415818 [34] and NCT00409188 [35]) in lung cancer. Currently, several Phase II clinical studies (NCT02134925 and NCT00773097) examined the efficacy of immunotherapies targeting tumor neoantigens against MUC1 in COAD. Thus, tumor-associated glycopeptide antigens of the MUC family may serve as ideal targets for the development of immunotherapeutic vaccines in COAD.

The human MUC family consists of at least 22 members: MUC1-MUC22 [36]. The aberrant expression of the MUC family has been reported to be a common feature of CRC. MUC1, MUC2, MUC4, and MUC6 are up-regulated in CRC, and their overexpression has been associated with disease progression [37–39]. However, the demethylation and down-expression of MUC5AC are predictive biomarkers for MSI and prognosis in CRC [38, 40–42]. A recent report presented a finding from multi-omics clinical data, including transcriptomics RNA-sequencing (mRNA, lncRNA, miRNA), DNA methylation, and gene mutations in the TCGA-STAD cohort demonstrated that MUC16 could be used as a potential biomarker to predict the response of immunotherapy and chemotherapy in gastric cancer [43], implying that MUC may be a broad-spectrum therapeutic target. However, an integrated bioinformatics analysis of MUC mutations in COAD across multiple databases has not been performed to date. Herein, in this study, we obtained neoantigens candidates of MUC mutant genes for COAD by the combination of a comprehensive analysis of public data and set up a prediction model for initiating the selection of high-confidence neoantigens derived from MUC mutations. We provided a series of potential candidates for tumor neoantigen-based immunotherapy in COAD.

## Methods

### Data collection and genetic mutation analysis

We procured somatic mutation data from 172 COAD samples within The Cancer Genome Atlas (TCGA), accessible through the official website (https://portal.gdc.cancer.gov/) and from 2070 COAD samples hosted on the cBioPortal, available at (https://www.cbioportal.org/). Additionally, the prognostic analysis results of overall survival (OS), disease-free survival (DFS), progression-free survival (PFS), and disease-specific survival (DSS) between the 19 MUC genes mutation group and the non-mutation group in COAD cancer patients were also conducted using data from these websites.

### Tumor-associated antigens prediction for MUC family

The Tumor Immune Estimation Resource 2.0 (TIMER2.0, http://timer.cistrome. org/) web server, a comprehensive resource integrating multiple immune infiltration estimation algorithms [44] was used for the analysis of tumor immunogenicity. The TISIDB web server, a web portal integrated with multiple heterogeneous data types, was used for tumor and immune system interaction analysis. A two-sided Wilcoxon rank-sum was employed to compare the expression levels of genes related to tumor-infiltrating immune cells, immunoinhibitors, immunostimulators, MHC molecules, chemokines, receptors and antitumor immunity between MUC family-mutated and wild-type groups.

### Neoantigen prediction

TSNAdb [27] collects 7748 tumor samples from TCGA and the Cancer Immunome Atlas (TCIA). We obtained the tumor-specific neoantigen data of 531 TCGA COAD samples from TSNAdb (http://biopharm.zju.edu.cn/tsnadb). Compared with NetMHCpan v2.8 [28, 30], NetMHCpan v4.0 [45] is trained based on both binding affinity data and mass spectrometry data, thus adopting stricter criteria for binding prediction. The binding affinities of wild-type and mutant peptides of the MUC family with the HLA alleles of patients were first predicted by the NetMHCpan (v2.8 and v4.0), algorithm in COAD using TSNAdb v1.0. Next, TSNAdb v2.0 combines the predicted results of DeepHLApan, MHCflurry, and NetMHCpan v4.0 for the identification of higher confidence neoantigens derived from SNV, INDEL, and Fusion [28]. To calculate DAI, MHC-I affinity was predicted for wildtype (WTA) and mutant (MA) peptide pairs arising from the same mutation and differing by a single amino acid. DAI was defined as WTA-MA for each peptide pair.

### Statistical analysis

Enumeration data including gene mutations were compared using the chi-square test or Fisher’s exact test, while measurement data like age, TMB, abundance of immune cells, and immune-associated gene expression were compared by Mann–Whitney U-test. Clinical outcomes were assessed using the Kaplan–Meier method and compared by the log-rank test. The TISIDB database was used to explore the correlations between MUC expression and immune- or molecular subtypes in COAD, in which a p-value < 0.05 was set as the satisfying criteria. To evaluate the predictive impact of MUC mutations on immunotherapy, we employed the 'IMvigor210CoreBiologies' package (version 1.0.0) within the R software, which encompasses clinical data of ICIs in patients with COAD. The TIMER database was employed for a comprehensive examination of tumor-infiltrating immune cells. In this analysis, the somatic copy number alterations (SCNAs) and mutation modules were evaluated using a two-sided Wilcoxon rank-sum test. The SCNAs module, as defined by GISTIC 2.0, includes the following categories: deep deletion (−2), arm-level deletion (−1), diploid/normal (0), arm-level gain (1), and high amplification (2). The value of p ≤ 0.05 was considered statistically significant.

## Results

### Exploring MUC genes mutations in colon adenocarcinoma datasets

**Fig. 1:**
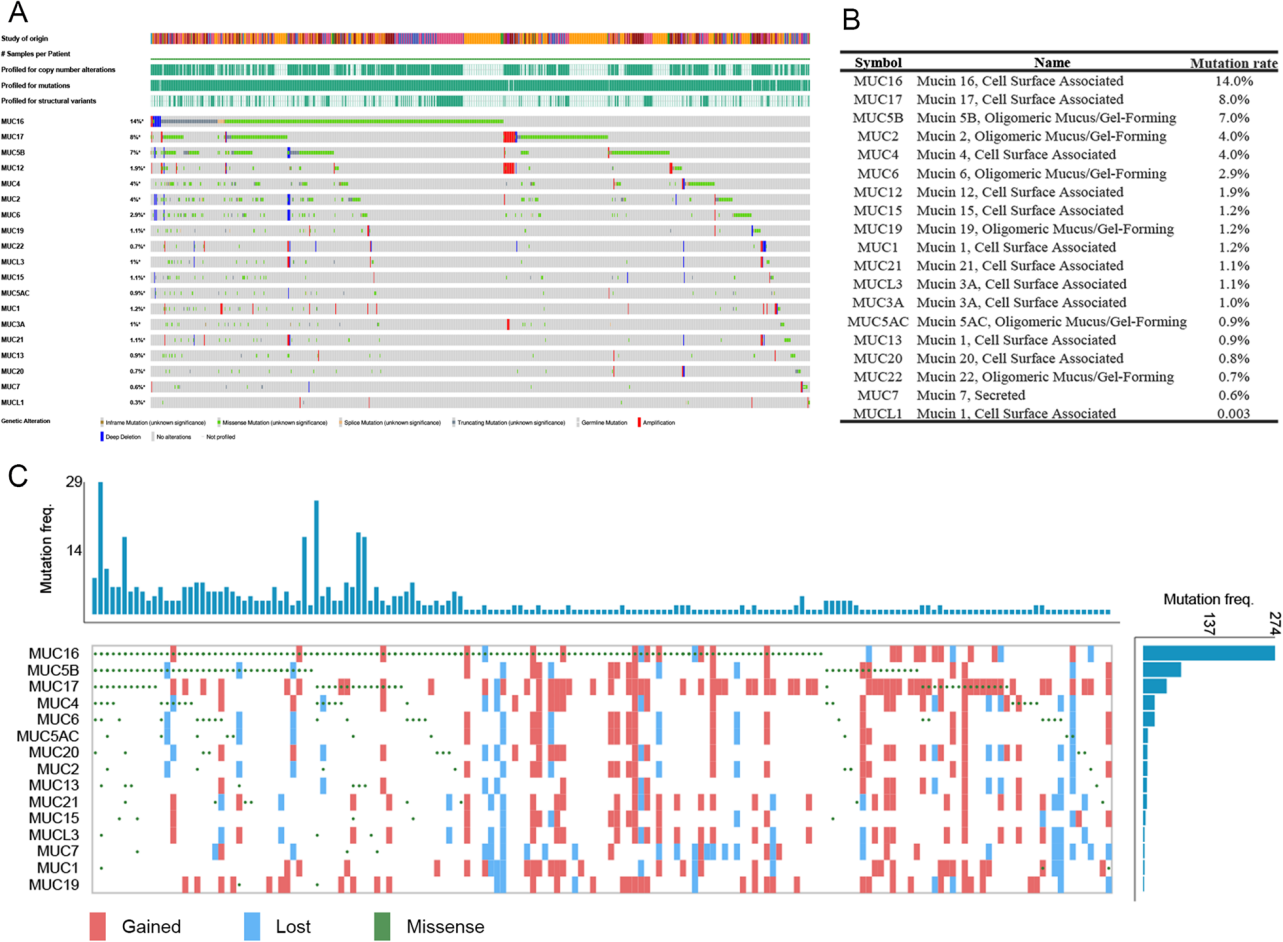
Interrogating the Mutational Landscape of MUC Genes in the Context of COAD Across Two Distinct Datasets. **A** Illuminating the Mutational Frequencies of Nineteen MUC Genes on cBioPortal website; **B** Providing a Tabular Representation of the Mutation Rates for these Genes; **C** The mutation frequencies of the 19 MUCs genes within the TCGA datasets

To identify potential tumor neoantigens in COAD, we determined the mutational frequencies of MUC genes within the context of a total of 2242 COAD across two distinct datasets (n1 = 2070, n2 = 172). Firstly, we present the mutational frequencies of nineteen MUC genes as cataloged on the renowned platform, cBioPortal (Fig. 1A, n1 = 2070). The genes in which recurrent mutations were frequently observed were MUC16 (14%), MUC17 (8%), MUC5B (7%), MUC2 (4%), MUC4 (4%), and MUC6 (2.9%), respectively. Within this framework, we present an exhaustive tabular representation detailing the mutation rates of these aforementioned genes (Fig. 1B). Continuing our analysis, we also explored the mutation frequencies of the same MUC genes using data from the TCGA datasets (Fig. 1C, n2 = 172). This search yielded similar results, further validating our findings from the cBioPortal platform. Further delving into the results, we observed distinct mutation patterns across the MUC genes. While MUC16 showed the highest mutation frequency at 75.4%, this notably high rate could be influenced by the small sample size and the randomness inherent in the data. This is followed by MUC5B at 22.8%, MUC17 at 13.5%, and MUC2 and MUC6 both at 8.7% (Additional file 6: Table S1). Through the analyses of these two databases, it becomes apparent that among the most prominent genes in terms of mutation frequency are MUC16, MUC17, MUC5B, MUC2, MUC4, and MUC6. These patterns highlight the varied roles and potential significance of these genes in cancer biology. It underscores the necessity for further research with a larger dataset to validate these findings, particularly for MUC16.

### Prognostic significance of MUC genes in COAD

**Fig. 2:**
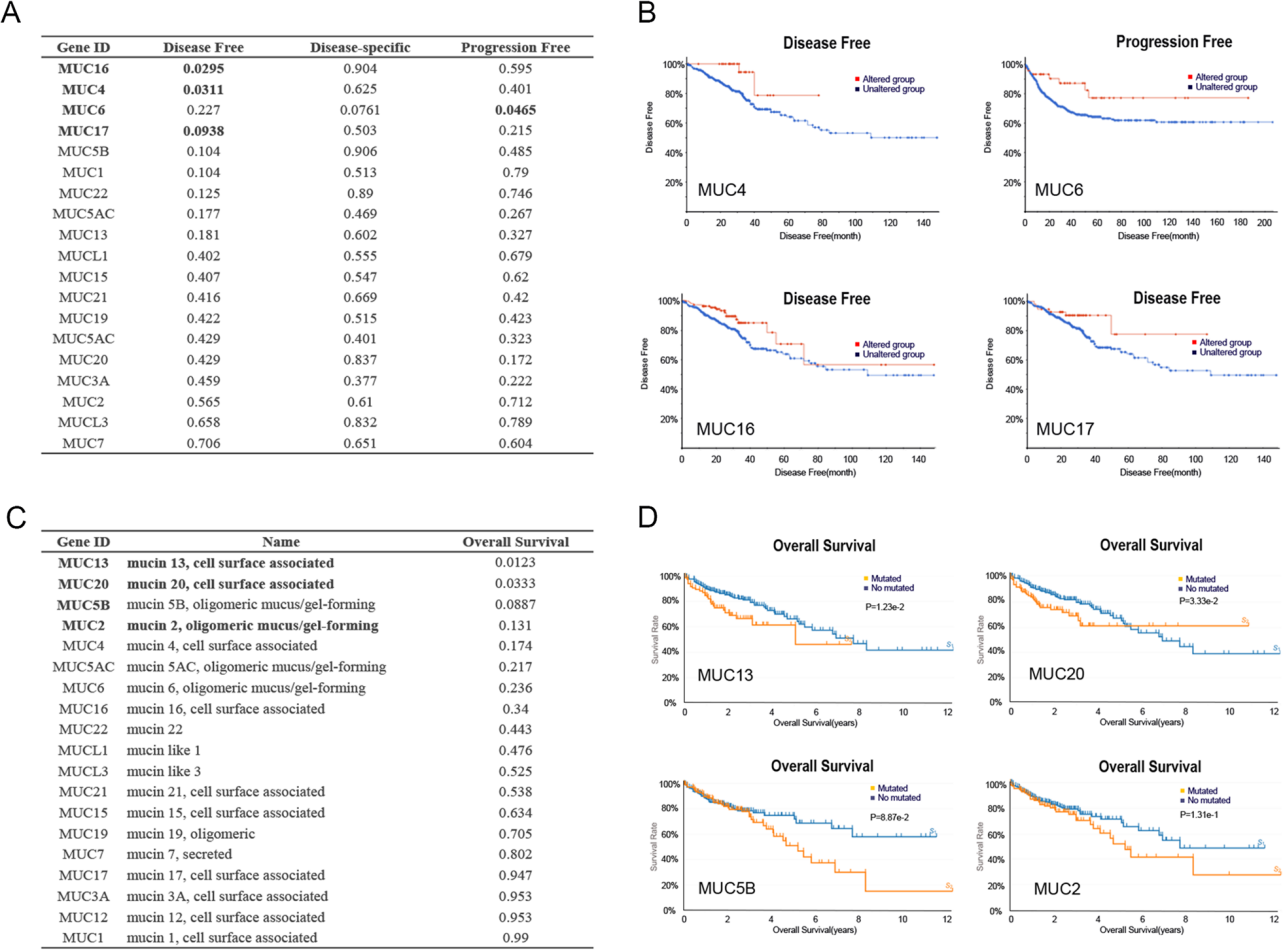
Revealing the Prognostic Significance of MUC Genes in two databases. **A** Exploring DFS, PFS, and DSS among COAD Patients, stratified between those with mutations in the 19 MUC genes and those without, as documented on the cBioPortal platform; **B** The Kaplan–Meier curve presents a portrayal of the four preeminent genes in COAD, characterized by their remarkable significance, as elucidated on the cBioPortal website; **C** The OS rate of the 19 MUCs genes mutation group and the non-mutation group in COAD cancer patients on the TCGA website; **D** The KM curve captures the top 3 genes with significant P value in the context of COAD within the TCGA website. DFS, Disease-Free Survival; PFS, Progression-Free Survival; DSS, Disease-Specific Survival; OS, Overall Survival

To investigate the MUC family that is functional as a potential candidate for mRNA vaccine targets, the prognostic significance of mutations in all MUC genes was analyzed in 2070 samples of COAD with survival data. We identified 3 genes associated with disease-free survival (DFS) (Fig. 2A), and 1 genes with progression-free survival (PFS) (Fig. 2A, B), respectively. Only MUC13, MUC20, and MUC5B were revealed to be closely associated with OS (Fig. 2C, D). In summary, our findings emphasize the crucial role of MUC genes associated with the prognoses of COAD patients, shedding light on the potential implications for clinical practice and therapeutic interventions.

### Exploring the correlation of MUC genes in the context of COAD

**Fig. 3:**
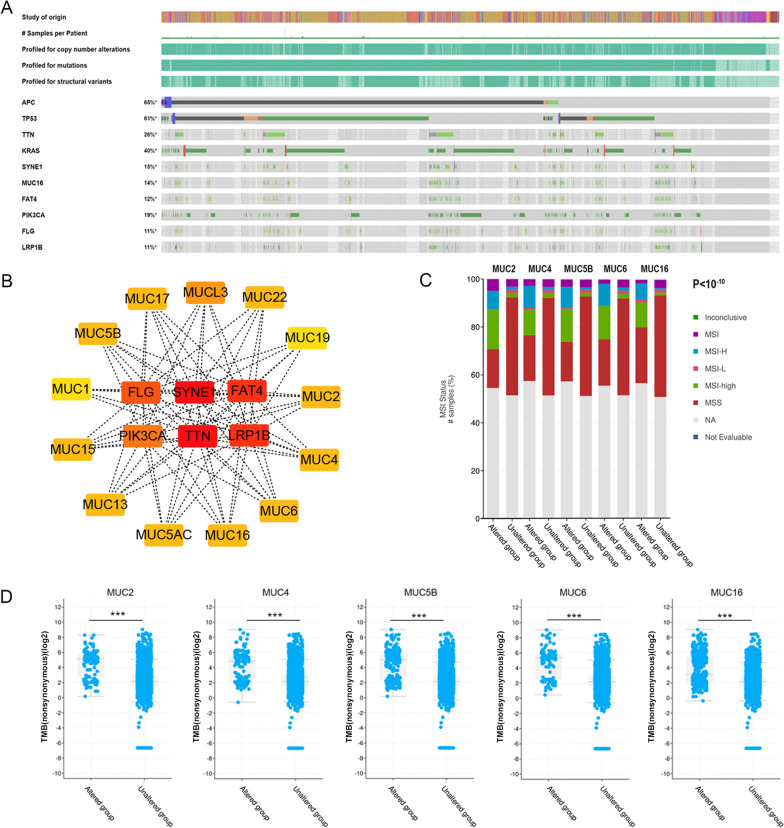
The correlation of MUCs with highly mutated genes and genetic instability. **A** The top ten genes with the highest mutation frequencies in COAD on the cBioPortal website; **B** The correlation of mutated MUC genes and the top ten highest mutation genes were analyzed by the cytoscope; **C** The relationship between the mutation status of MUC genes and MSI (MUC16, MUC2, MUC4, MUC5B, and MUC6); **D** The plots showing the association between TMB and mutation status of five MUC genes (MUC2, MUC4, MUC5B, MUC6 and MUC16) frequently mutated in the COAD cohort. MSI, Microsatellite Instability; TMB, Tumor Mutational Burden

We analyzed the correlation of MUC genes with clinical factors within the context of COAD. Initially, we carefully identified the top ten genes with the highest mutation frequencies in COAD using the cBioPortal website, as depicted in Fig. 3A. Subsequently, we conducted a detailed analysis to explore the intricate interaction between mutated MUC genes and these top ten genes. In COAD, the identified top ten most frequently mutated genes were APC, TP53, TTN, KRAS, SYNE1, MUC16, FAT4, PIK3CA, FLG, and LRP1B (Fig. 3A). Among these, a subset comprising SYNE1, FAT4, PIK3CA, FLG, and LRP1B demonstrated a significant correlation with mutations in the MUC gene family, which includes MUC1, 2, 3, 4, 5AC, 5B, 6, 13, 15, 16, 17, 19, and 22. Furthermore, we analyzed the relationship between the mutation status of MUC genes and genetic MSI and TMB (Fig. 3C). Our investigation found a strong correlation between the mutation status of five specific MUC genes and TMB (MUC2, MUC4, MUC5B, MUC6 and MUC16) (Fig. 3D). The relevance between MUC mutation and TMB/TSI implied the potential effect of MUC mutation on tumor progression and immunotherapy.

### Immunological correlation of MUC family mutation

**Fig. 4:**
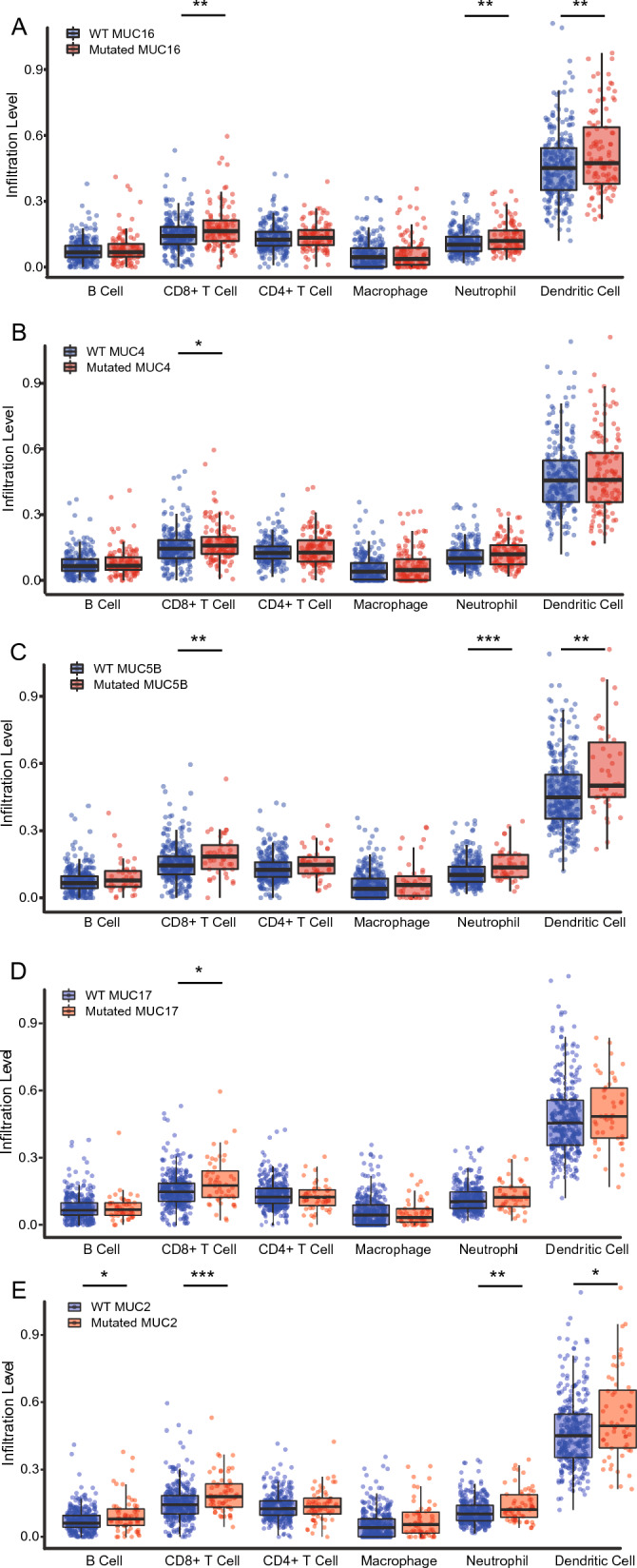
Five MUC genes comprehensive analysis of tumor-infiltrating immune cells (B cell, CD8^+^ T cell, CD4^+^ T cell, Macrophage, Neutrophil, Dendritic cell) in COAD using the Timer database. **A** MUC16; **B** MUC4; **C** MUC5B; **D** MUC17; **E** MUC2. The mutation module compares the levels of immune infiltrates with or without the presence of a given mutation. Box plots are generated for each immune subset, to compare the distributions of immune infiltration levels under different gene mutation statuses, with statistical significance estimated using a two-sided Wilcoxon rank-sum test

**Fig. 5:**
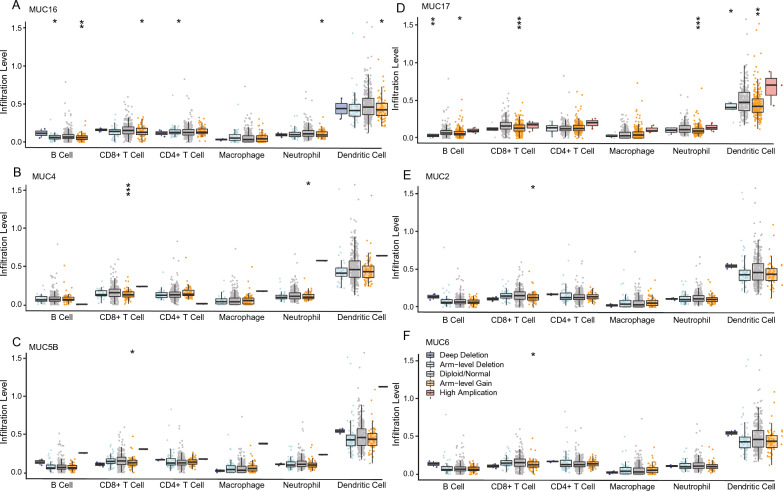
SCNA module provides the comparison of tumor infiltration levels among tumors with different somatic copy number alterations for a given gene. SCNAs are defined by GISTIC 2.0, including deep deletion (−2), arm-level deletion (−1), diploid/normal (0), arm-level gain (1), and high amplification (2). Box plots are presented to show the distributions of each immune subset at each copy number status in COAD, i.e., **A** MUC16; **B** MUC4; **C** MUC5B; **D** MUC17; **E** MUC2; **F** MUC6. The infiltration level for each SCNA category is compared with the normal using a two-sided Wilcoxon rank-sum test

To depict the immunological role of MUC mutation comprehensively that targeting MUC mutation might benefit from immunotherapy in COAD, we investigated the association between MUC family mutation and tumor microenvironment (TME) and immune subtype. The cancer-associated immune cells were related to MUC family mutation in COAD (Fig. 4A–E). We assessed the enrichment scores of MUC family mutated- and wildtype- cohorts in immune cells i.e., CD8^+^ T cells, CD4^+^ T cells, dendritic cells, myeloid-derived suppressor cells (MDSCs), macrophages, B cells, neutrophil cells, NK, and Treg cells in COAD. Furthermore, the enrichment scores within the different cohorts of MUC copy number variation (CNV) showed significant differences (Fig. 5A–E, Additional file 1: Fig. S1, Additional file 2: Fig. S2, Additional file 3: Fig. S3 and Additional file 4: Fig. S4) in immune cells, including CD8^+^ T cells, CD4^+^ T cells, dendritic cells, neutrophil cells, Th2 cells, B cells, and NKT. Comprehensive analyses of above data about the associations of MUCmuts in the top six mutated genes with different immune parameters, we found the common points, that the mutations of the MUC family i.e., 16, 17, 5B, 4, 2, and 6 were immunogenic to patients, including higher immune scores and more immune cells, especially CD8^+^ T cells.

**Fig. 6:**
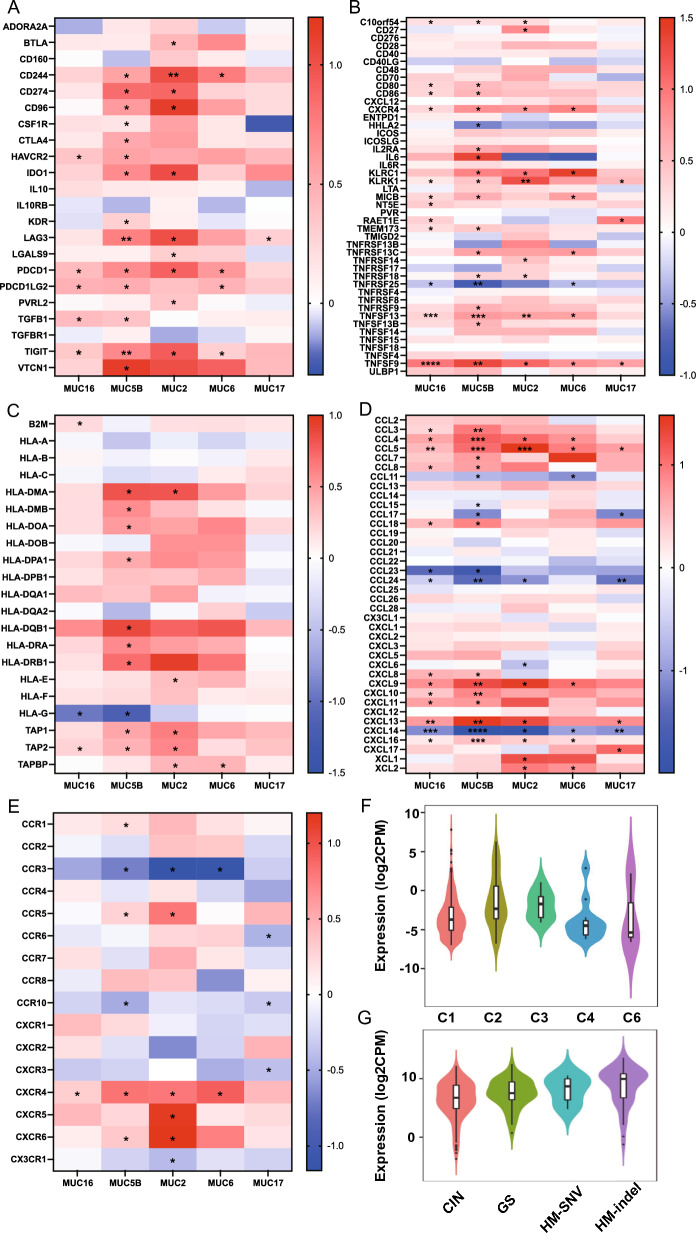
Immune and molecular correlation with the mutation or expression of MUC family. **A** immunoinhibitors; **B** immunostimulators; **C** MHC molecules; **D** chemokine; **E** receptor. These immunomodulators were collected from Charoentong's study. **F** MUC16 expression was high associated with immune and molecular subtypes, respectively in COAD, Kruskal–Wallis Test: P value = 1.96e−06; n = C1 332, C2 85, C3 9, C4 12, C6 3; **G** MUC 5B expression was high associated with immune and molecular subtypes, respectively in COAD, Kruskal–Wallis Test: P value = 9.5e−08; n = CIN 226, GS 49, HM-SNV 6, HM-INDEL 60

The immune checkpoint (ICP) genes were found to play a role in immune cell infiltration and immunotherapy outcomes [43, 46]. Our result indicates that MUC family gene mutation was positively related to the expression of these ICP genes in COAD: i.e. immunoinhibitors including CD244, CD274, CD96, CTLA4, HAVCR2, IDO1, LAG3, PDCD1, PDCD1 LG2, TGFβ1 and TIGIT etc. in COAD (Fig. 6A); immunostimulators including C10orf54, CXCR4, KLRC1, KLRK1, MICB, TNFSF13, and TNFRSF25 (Fig. 6B); MHC molecules including HLA-DMA, HLA-G, Transporter associated with antigen processing (TAP)1, TAP2, TAPBP etc. (Fig. 6C); chemokine including CCL4, CCL5, CCL24, CXCL9, CXCL13, CXCL14 and CXCL16 (Fig. 6D); receptor including CCR3 and CXCR4 (Fig. 6E). Additionally, high MUC16 expression was also promoted in the C1 (wound healing) immune subtypes and MUC5B in CIN molecular subtypes (Fig. 6F, G), which indicated that MUC mutated genes may be involved in TME remodeling. Since TMB, MSI, and NEO are considered predictors for response to tumor immunotherapy within the TME [47, 48], our above-analyzed data showed a positive association of TMB and MSI with MUCmut in COAD (Fig. 3C, D). Collectively, MUC family mutation may affect antitumor immunity through its association with immune infiltrating cells, ICPs, MSI, and TMB in COAD tissues. These findings suggested that peptides from MUCmut might be potential neoantigens for COAD.

### Characteristics of neoantigens of MUCmut in COAD

**Fig. 7:**
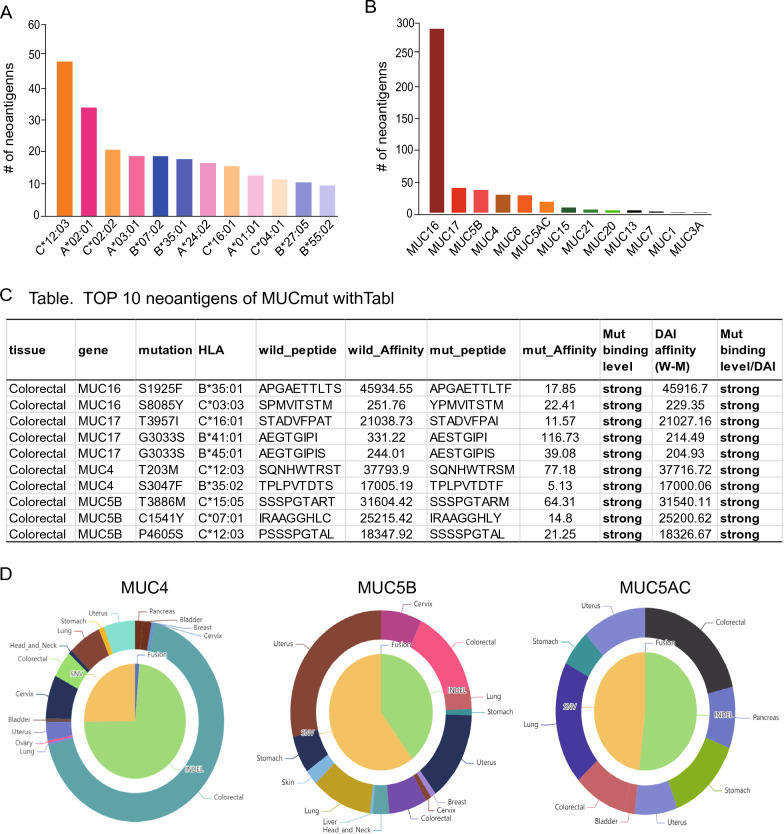
Predicted neoantigens of mutated MUC family for COAD The top 12 HLA alleles **A** in MUCmut **B** with the number of predicted neoantigens are displayed in COAD, with the detailed neoantigen information listed **C** The binding level ‘Strong’ indicates strong binding with IC50 < 150 nM based on Mutate binding level or DAI > 150 nM. **D** The INDEL mutational signature of MUCmut in COAD

To discover the potential neoantigens profiling of mutated genes in COAD, we comprehensively analyzed all mutations of the MUC family in 531 COAD samples obtained from the TSNAdb database [27] using the method of DAI to select high-confidence TSA. We first confirmed that MUC16 was ranked as the top 6 most frequently mutated genes (Fig. 3A), and was moved to the top 2 generate-neoantigens across all mutated genes in COAD (Additional file 5: Fig. S5). Next, 1243 SNVs of MUCmut neoantigens by TSNAD v1.0 NetMHCpan v2.8, and 452 SNVs of MUCmut neoantigens by TSNAD v1.0 NetMHCpan v4.0 were identified (Additional file 7: Table S2). The order for the number of SNV neoantigens was 282 for MUC16mut, 38 for MUC17mut, 35 for MUC5mut, 28 for MUC4mut, 27 for MUC6mut and 17 for MUC5ACmut (Fig. 7B). The two most binding HLA alleles of MUCmut neoantigens were HLA-C*12:03 and HLA-A*02:01 (Fig. 7A), which also ranked as the top 2 HLA alleles of predicted neoantigens in COAD by TSNAdb1.0/NetMHCpan v2.8 (Additional file 5: Fig. 5B). Next, we use TSNAdb v2.0 to identify higher confidence neoantigens of MUCmut. The results showed that 325 potential neoantigens of MUC mutated genes involving 157 INDELs, 1 TS, and 57 SNVs were identified (Additional file 8: Table S3). Among 157 INDELs, the top 3 MUCmut neoantigens were MUC4, 5B, and 5AC (Fig. 7D and Addition file 7: Table S2). Moreover, we applied the tool of DAI to select the higher-confidence affinity neoantigens and obtained 10 high-confidence SNV MUCmut neoantigens out of 57 SNVs (Fig. 7C).

## Discussion

Identification of immunogenic neoantigens from numerous sources is a crucial step in the development of effective immunotherapies [23]. However, the reported studies of the identification of potential neoantigens in COAD are rare. To address whether the MUC family can potentially target tumor-specific immunotherapy in COAD, we performed a thorough screen across the MUCmut and a detailed analysis of immune infiltration estimation algorithms of MUCmut in COAD. After deep analysis, our data revealed the potential value of MUC mutated genes as a predictor of prognosis and immunological biomarkers. Next, we performed a typical workflow for neoantigen prediction as following steps: (i) mutation calling, (ii) HLA typing, (iii) neoantigen filtering and prioritization based on HLA binding affinity. Through our analysis with two neoantigen-related databases (TSNAdb and NetMHCpan) plus DAI, we obtained 10 high-confidence SNVs and 157 INDELs neoantigens from 6 MUC mutate genes (MUC16, MUC17, MUC4, MUC6, MUC2, and MUC5B) that may serve as candidate targets for neoantigen vaccines.

Currently, the initial stage in the process of detecting possible neoantigens from NGS data is mapping tumor-specific genetic abnormalities using WES of the tumor and normal DNA. In this study, with the integrated analysis of the 6261 COAD samples, somatic genomic alterations for the MUC family include SNVs and gene fusions which might promote the production of tumor neoantigens. The top six mutated family members were MUC16, MUC17, MUC5B, MUC2, MUC4, and MUC6 with the mutation frequencies from 14 to 2.9%. The high mutation of MUC16, MUC4 and MUC6 had a strong association with PFS or DFS in COAD, which was consistent with previous studies for MUC16 [49] and MUC4 [50].

Currently, multiple potential factors have been identified for predicting the clinical outcome of immunotherapy, including the tumor mutation burden (TMB) [51], MSI status [7], neoepitope load [52–55], PD-L1 level [55], CD8^+^ T-cell density [56], interferon-γ gene signature [57], and MHC and T-cell receptor repertoire [58, 59]. The TMB and MSI were applied as clinical biomarkers for a potential response to ICI immunotherapy in colorectal and other solid tumors [51]. Our study showed a strong association between several MUC-mutated genes with TMB and MSI in COAD. This finding was in line with previous studies, showing that the cancer-associated mucins play a role in immune modulation and metastasis [60]. Tumor-infiltrating lymphocytes in the tumor microenvironment have been indicated to be efficient in predicting prognosis and immunotherapeutic efficacy for cancer [58]. In the present study, we found that the infiltration levels of several effector TIICs, such as activated CD8^+^ T cells, activated CD4^+^ T cells, activated B cells, T Neutrophil cells, dendritic cells, and T helper cells, were significantly upregulated in COAD patients with MUC mutated genes. In addition, ICB targeting these ICPs, such as PD1-blocking antibodies, pembrolizumab, and nivolumab, have shown efficacy in patients with metastatic MSI-H CRC, and they have been granted accelerated FDA approval [7]. Our study showed a strong relationship between MUC mutated genes and key ICPs, such as CD274 (PD-L1), CTLA4, HAVCR2, IDO1, LAG3, PDCD1, TGFβ1and TIGIT, as well as MSI and TMB in COAD. This indicates that it can be of value in further investigations on developing novel immunotherapeutic strategies as tumor neoantigens to target MUC mutated genes, which might benefit COAD patients. Intriguing, cancer mutations can form neo-epitopes recognized by T cells on HLA molecules, which contributes to the clinical success of immunotherapy [18, 52, 61–63], and creates enthusiasm for neo-epitope vaccines [14, 64]. In our analyses, MHC molecules include HLA-DMA, HLA-G, TAP1, TAP2, and TAPBP. were highly related to identified mutations of the MUC family.

Based on analyses with TSNAD v1.0 NetMHCpan v4.0 [28, 65, 66], we obtained 452 SNV unique neoantigens involving 13 MUC genes in 531 COAD patients (Additional file 6: Table S1). Recent clinical studies have shown a correlation between the burden of strong-binding neopeptides with an affinity for MHC-I of < 500 nM (referred to as neoantigens) and patient outcomes in advanced melanoma and lung cancer [52, 61, 62]. After we defined the binding level ‘Strong’ indicates strong binding with IC50 < 150 nM based on Mutate binding level or ‘Weak’ indicates weak binding with 150 nM < IC50 < 500 nM, we obtained 271 ‘Strong’ neoantigens and 181 ‘Weak’ neoantigens of MUC. Additionally, an important future field is to discovery of neoantigens created by cancer-specific INDELs (insertions and deletions), fusion genes, and splice variants that have a lower degree of similarity to self-antigens than SNV-derived neoantigens.

Similar to SNV-encoded neoantigens, INDEL encoded neoantigens are more common in cancers with MSI-H, which is determined by the absence of DNA mismatch repair (MMR) mechanisms. As the evolution of MMR-deficient cancers is mainly triggered by mutations that inactivate tumor suppressor genes containing coding microsatellites, frameshift peptide neoantigens are more frequently shared among MMR-deficient cancers than missense mutation-derived neoantigens [67]. In this study, through comprehensive analyses by TSNAD v2.0 NetMHCpan v4.0, 157 INDELs, 1 TS, and 57 SNVs were identified as potential neoantigens for MUC mutated genes showing high affinity with MHC-I. Interestingly, 94 INDELs were for MUC4, which is consistent with a previous study that found that MUC4 was highly alternative splicing in CRC [68].

Mechanistically, the difference in predicted affinity for any paired wild-type/mutant peptides termed as differential agretopicity index (DAI) is a broad and better indicator of neuropeptide dissimilarity from self and a feature of immunogenicity [69–71]. In this study, among TSNAD v2.0 NetMHCpan v4.0 analyzed data, of 59 SNVs, 10 mutant neopeptides selected in the group of both binding level ‘Strong’ and DAI strong were 2 MUC16, 3 MUC17, 3 MUC5B, 2 MUC4. In Prof. Rosenberg’s clinical study [13] on the identification of tumor neoantigen using reactive tumor-infiltrating lymphocytes from 75 patient gastrointestinal cancers, one patient of MUC4 mutant neoantigen was recognized by CD8^+^ T cells and another patient with MUC6 mutant neoantigen were recognized by CD4^+^ T cells. All these results greatly expand the universe of target cancer antigens and identify new tools for anti-MUC immunotherapy in COAD.

## Conclusion

In conclusion, we identified neoantigen candidates of MUC mutant genes in COAD by the combination of a comprehensive analysis and set up a prediction model for initiating the selection of high-confidence neoantigens derived from MUC mutations. These candidates could be potential targets for neoantigen-based immunotherapy. The validation for the strong immunogenic effects of these potential antigens should be further evaluated in a large patient cohort before being utilized in clinical setting. This study could promote neoantigen-based immunotherapy for broader application in COAD.

### Supplementary Information

**Additional file 1: Figure S1.** Relations between the abundance of tumor-infiltrating lymphocytes (TILs) and the mutation of MUC16. The immune-related signatures types from Charoentong's study, the relative abundance of TILs in COAD was inferred by using gene set variation analysis (GSVA) based on gene expression profile.

**Additional file 2: Figure S2**. Relations between the abundance of TILs and the mutation of MUC5B. The immune-related signatures types from Charoentong's study, the relative abundance of TILs in COAD was inferred by using GSVA based on gene expression profile.

**Additional file 3: Figure S3.** Relations between the abundance of TILs and the mutation of MUC17. The immune-related signatures types from Charoentong's study, the relative abundance of TILs in COAD was inferred by using GSVA based on gene expression profile.

**Additional file 4: Figure S4.** Relations between the abundance of TILs and the mutation of MUC6. The immune-related signatures types from Charoentong's study, the relative abundance of TILs in COAD was inferred by using GSVA based on gene expression profile.

**Additional file 5: Figure S5.** The top 20 genes and HLA alleles with the number of predicted neoantigens are displayed in COAD by TSNAdb1.0/NetMHCpan v2.8.

**Additional file 6: Table S1.** Mutation Rates of MUC family within the TCGA Dataset.

**Additional file 7: Table S2.** MUCmut neoantigens predicted by TSNAD v1.0 /NetMHCpan v2.8.

**Additional file 8: Table S3. **MUCmut neoantigens by TSNAD v2.0/NetMHCpan v4.0.

## Data Availability

The datasets used and/or analyzed during this study are available from the corresponding author upon reasonable request.

## Abbreviations

COAD: Colon adenocarcinoma
SNVs: Single nucleotide variants
CNV: Copy number variation
TMB: Tumor mutation burden
TSI: Tumor-specific immunoreactivity
TME: Tumor microenvironment
ICP: Immune checkpoint
dMMR: Deficient mismatch repair
ICIs: Immune-checkpoint inhibitors
CRC: Colorectal cancer
pMMR: Proficient mismatch repair
MSI-L: Microsatellite instability-low
TAP: Transporter associated with antigen processing
MHC: Major histocompatibility complex
OS: Overall survival
DFS: Disease-free survival rate
PFS: Progression-free survival rate
DSS: Disease-specific survival rate
TCIA: The Cancer Immunome Atlas
TME: Tumor microenvironment
MDSCs: Myeloid-derived suppressor cells
