# Supplementary figures and images for: Characterizing and forecasting neoantigens-resulting from MUC mutations in COAD
Source: J Transl Med. 2024 Mar 27;22:315. doi: 10.1186/s12967-024-05103-z (PMC10967086; doi:10.1186/s12967-024-05103-z)

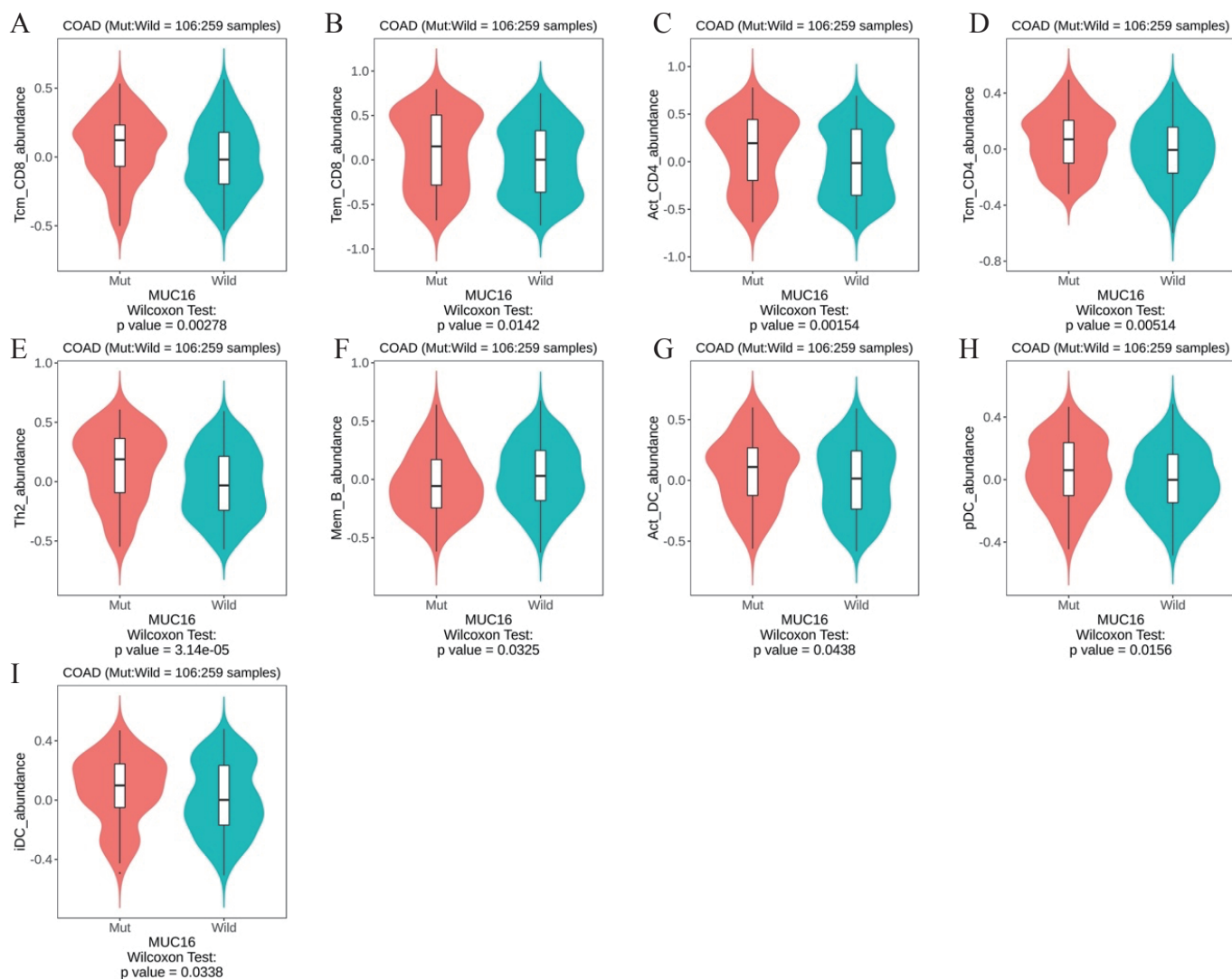

Supplement: Supplementary file 1 — Additional file 1: Figure S1. Relations between the abundance of tumor-infiltrating lymphocytes (TILs) and the mutation of MUC16. The immune-related signatures types from Charoentong's study, the relative abundance of TILs in COAD was inferred by using gene set variation analysis (GSVA) based on gene expression profile. [file 12967_2024_5103_MOESM1_ESM.pdf]

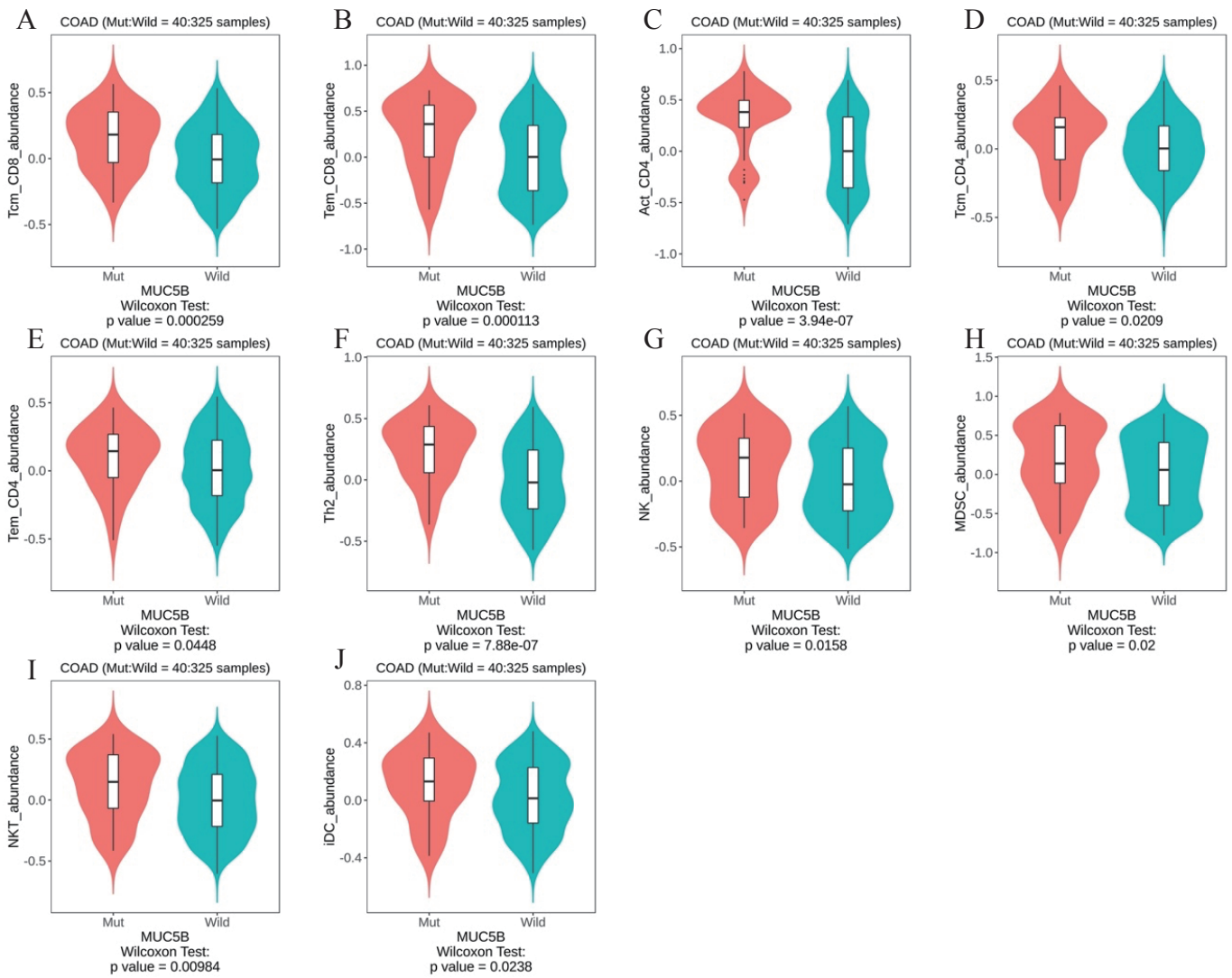

Supplement: Supplementary file 2 — Additional file 2: Figure S2. Relations between the abundance of TILs and the mutation of MUC5B. The immune-related signatures types from Charoentong's study, the relative abundance of TILs in COAD was inferred by using GSVA based on gene expression profile. [file 12967_2024_5103_MOESM2_ESM.pdf]

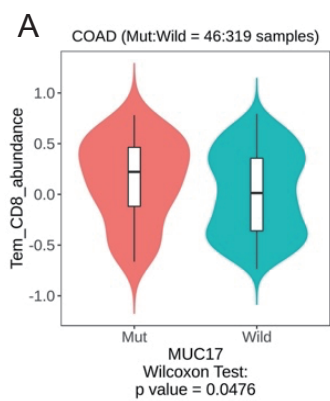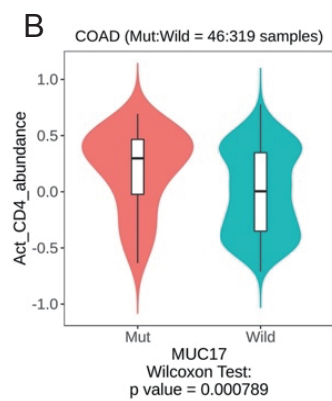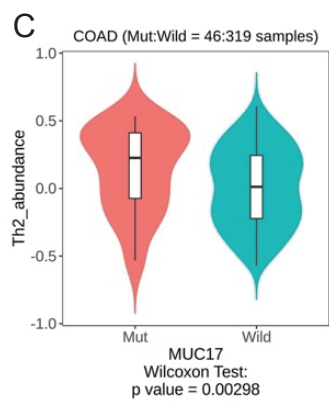

Supplement: Supplementary file 3 — Additional file 3: Figure S3. Relations between the abundance of TILs and the mutation of MUC17. The immune-related signatures types from Charoentong's study, the relative abundance of TILs in COAD was inferred by using GSVA based on gene expression profile. [file 12967_2024_5103_MOESM3_ESM.pdf]

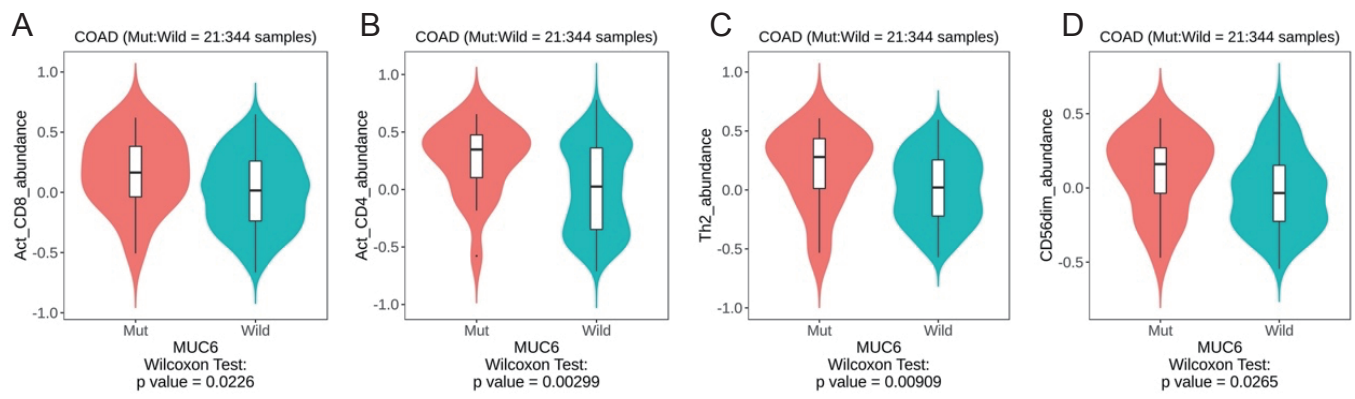

Supplement: Supplementary file 4 — Additional file 4: Figure S4. Relations between the abundance of TILs and the mutation of MUC6. The immune-related signatures types from Charoentong's study, the relative abundance of TILs in COAD was inferred by using GSVA based on gene expression profile. [file 12967_2024_5103_MOESM4_ESM.pdf]

A

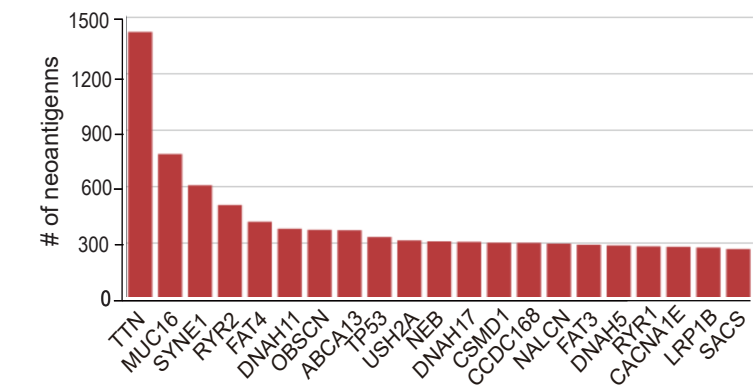

B

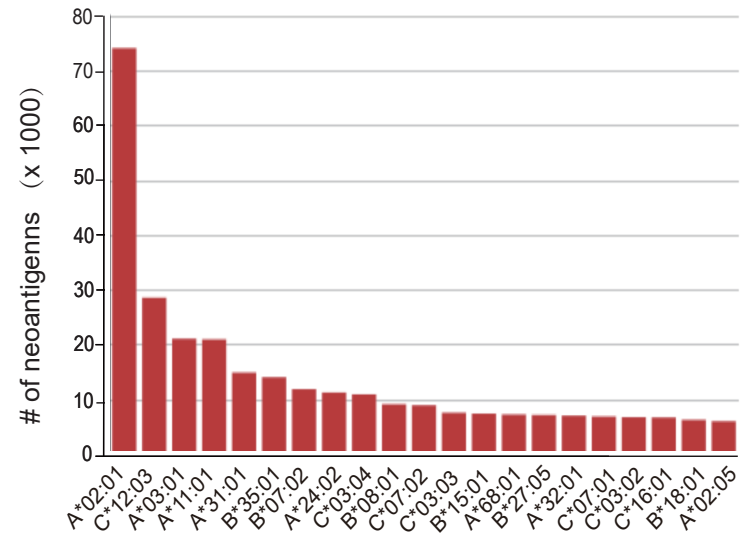

Supplemental Figure 5

Supplement: Supplementary file 5 — Additional file 5: Figure S5. The top 20 genes and HLA alleles with the number of predicted neoantigens are displayed in COAD by TSNAdb1.0/NetMHCpan v2.8. [file 12967_2024_5103_MOESM5_ESM.pdf]
